# Provider and patient perspectives on opioids and alternative treatments for managing chronic pain: a qualitative study

**DOI:** 10.1186/s12875-016-0566-0

**Published:** 2017-03-24

**Authors:** Lauren S. Penney, Cheryl Ritenbaugh, Lynn L. DeBar, Charles Elder, Richard A. Deyo

**Affiliations:** 1 South Texas Veterans Health Care System, 7400 Merton Minter Blvd, San Antonio, TX 78229 USA; 2 The University of Arizona, Department of Family and Community Medicine, 1450N. Cherry Ave, Tucson, AZ 85719 USA; 3 Kaiser Permanente Center for Health Research Northwest Region, 3800N. Interstate Ave, Portland, OR 97227 USA; 4 Oregon Health & Science University, 3181S.W. Sam Jackson Park Rd, Portland, OR 97239 USA

**Keywords:** Chronic pain, Primary care, Opioids, Complementary medicine, Acupuncture, Chiropractic

## Abstract

**Background:**

Current literature describes the limits and pitfalls of using opioid pharmacotherapy for chronic pain and the importance of identifying alternatives. The objective of this study was to identify the practical issues patients and providers face when accessing alternatives to opioids, and how multiple parties view these issues.

**Methods:**

Qualitative data were gathered to evaluate the outcomes of acupuncture and chiropractic (A/C) services for chronic musculoskeletal pain (CMP) using structured interview guides among patients with CMP (*n* = 90) and primary care providers (PCPs) (*n* = 25) purposively sampled from a managed care health care system as well as from contracted community A/C providers (*n* = 14). Focus groups and interviews were conducted patients with CMP with varying histories of A/C use. Plan PCPs and contracted A/C providers took part in individual interviews. All participants were asked about their experiences managing chronic pain and experience with and/or attitudes about A/C treatment. Audio recordings were transcribed and thematically coded. A summarized version of the focus group/interview guides is included in the Additional file 1.

**Results:**

We identified four themes around opioid use: (1) attitudes toward use of opioids to manage chronic pain; (2) the limited alternative options for chronic pain management; (3) the potential of A/C care as a tool to help manage pain; and (4) the complex system around chronic pain management. Despite widespread dissatisfaction with opioid medications for pain management, many practical barriers challenged access to other options. Most of the participants’ perceived A/C care as helpful for short term pain relief. We identified that problems with timing, expectations, and plan coverage limited A/C care potential for pain relief treatment.

**Conclusions:**

These results suggest that education about realistic expectations for chronic pain management and therapy options, as well as making A/C care more easily accessible, might lead to more satisfaction for patients and providers, and provide important input to policy makers.

**Trial registration:**

ClinicalTrials.gov NCT01345409, date of registration 28/4/2011

**Electronic supplementary material:**

The online version of this article (doi:10.1186/s12875-016-0566-0) contains supplementary material, which is available to authorized users.

## Background

Chronic pain conditions are pervasive public health issues that exact a staggering human and economic toll. Indeed, persistent pain affects an estimated 19% of adults in the U.S. [1] and results in $560 billion in direct medical treatment costs and lost productivity. This is especially troubling as these conditions disproportionately affect vulnerable populations [2]. The past decades have seen a dramatic escalation in the use of prescription opioids for the treatment of chronic non-cancer pain [3]. The period spanning the 1980’s through the early 2000’s has been widely described as an opioid epidemic [4] driven by the rising number of chronic non-cancer pain sufferers, liberalization of laws governing the use of opioids in pain treatment, aggressive pharmaceutical marketing, an acceptance of opioids as standard treatment of chronic pain, and mounting pressure on physicians to avoid undertreating pain [3, 5–7].

Along with the rise in opioid prescribing for pain treatment [8] has come an increasing awareness of adverse outcomes, including addiction, transitioning to illegal drugs, and death (e.g., [9–14]) as well as concern about the limitations and efficacy of treatment with opioids in chronic pain management [7, 15–17]. In response, recent guidelines have been proposed for mitigating the risk of adverse outcomes [18], but evidence of effectiveness of many such guidelines is lacking [19]. Further, the clinical milieu has fostered a shift toward treatment that focuses on increasing patients’ functioning rather than on pain reduction [20].

Redirecting patients with chronic pain from opioid therapy toward behavioral and other non-pharmacologic modalities has proven challenging. This is partly due to the paucity of comprehensive chronic pain management programs [21]. Additionally, only a few high-quality studies have assessed the effectiveness of interventions to taper off prescribed opioid use [22, 23]. While primary care providers (PCPs) are often aware of the risks and limitations of prescribing opioids as a monotherapy for pain, primary care practices often lack readily available systematic, integrated, and interdisciplinary treatment options [24, 25] or support for making prescribing decisions [26]. Both physicians and their patients are frustrated when alternatives to opioid therapy or tapering off opioid therapy are needed but effective and accessible alternatives are lacking.

Although other non-pharmacotherapy options exist [2, 27, 28], complementary and alternative medicine (CAM) may offer important non-opioid options for patients with chronic pain. In the United States, CAM treatment is used far more often for chronic musculoskeletal pain (CMP) than for any other condition [29]. A recently published survey of patients with chronic pain at a large health maintenance organization, for example, reported that 58% of respondents had used acupuncture, chiropractic care, or both [30]. Among CAM treatments for CMP, acupuncture and chiropractic (A/C) care are the most highly accepted by physician groups [31, 32] even though such treatments are only modestly efficacious [33–36]. Nevertheless, the potential for adverse outcomes is low [37] and the treatment could facilitate self-care lifestyle changes considered instrumental to improve longer-term functioning for patients with CMP [38, 39].

While much of the current literature has focused on the problems associated with opioid medications, a growing body of work is examining how to mitigate risks of adverse outcomes and increase the potential effectiveness of other pain therapies. Despite this literature, little has been written about the practical issues providers and patients face when trying to access alternatives to opioids for managing chronic pain and how both patients and their various providers view these issues. This paper fills this substantial gap in the literature by describing and comparing patient, PCP, and A/C providers’ perspectives of the treatment of chronic pain as a complex system in the context of their experiences accessing alternatives to opioids.

The goal of our analysis was to inform current debates about chronic opioid use for pain management and to describe the barriers providers face in changing therapeutic practice and CMP patients face in using such practices and co-managing their needs.

## Methods

### Design

The data for this paper were gathered during the early phases of a multi-phase, mixed-methods study to evaluate the outcomes of A/C services for CMP [40]. This paper uses data from focus groups and interviews with PCPs, patients with chronic pain, and acupuncturists and chiropractors to describe the challenges these groups face when opioids are the primary treatment available for chronic pain management but patients and health care providers alike seek other options. This paper further describes the context of opioid pain medication use in one large integrated health plan from the perspective of these patients and health care providers and how the attitudes, beliefs, practices, and systems may inadvertently facilitate and maintain the use of opioids. The substudy described here was conducted between September 2012 and June 2013.

### Setting

This study was conducted within a large managed care organization in the Pacific Northwest (Kaiser Permanente Northwest – KPNW). Nearly all of KPNW’s approximately 530,000 members have a chiropractic care benefit, and most (with the exception of Medicare patients) have an acupuncture benefit. Patients with musculoskeletal pain can be referred by a PCP or specialty physician to a contracted acupuncturist or chiropractor for a limited number of visits when clinically indicated. After referrals are vetted by the health plan’s referral office, patients make their own appointments with their chosen A/C clinicians.

### Participants

This paper is based on upon qualitative data from 129 participants. This includes health care system plan members with CMP who had or had not used A/C therapy (*n* = 90), PCPs who referred patients to A/C care (*n* = 25), and contracted community A/C providers who treated a high volume of managed KPNW’s CMP patients (*n* = 14). An accurate and more detailed discussion of the overall project methods and participant recruitment for this qualitative phase can be found in the overall project design paper [41]. We recorded and transcribed all interviews and focus groups.

Health plan members with CMP, who had answered a plan survey [30] indicating both A/C use and a willingness to participate in focus groups or interviews, were recruited through postal mail. Our preferred method of patient data collection was focus groups, as this has proved at our center to be a mechanism that is popular among patients and particularly useful for gathering information on patient attitudes and experience with the health plan. Eighty plan members participated in 11 focus groups, which were composed based on acupuncture use (yes/no), chiropractic use (yes/no) and self-pay for or referral to A/C. Plan members unable to make the scheduled focus groups but in undersubscribed cells (*n* = 10) were recruited to individual interviews covering the same material. Four hundred and forty-three letters were sent. Out of those who responded, 63 patients refused, 90 agreed, and the remainder was not further contacted because the interview/focus group slots filled. Focus groups and interviews took place in private rooms within health plan facilities. Overall patient demographics for the survey are provided in Elder et al. [30].

### Interviews and focus groups

Interview and focus group guides were developed by staff and investigators using an iterative process. Original drafts were based on the well-accepted “behavioral models” for health care decision-making [42, 43] and integrated CAM services [44]. Questions were adjusted slightly over the course of the study to explore emerging issues. The interview guides ranged between 13 and 24 questions and we allowed 120 min for the focus groups and 30–45 min for each interview.

Three highly experienced members of the study team conducted interviews and focus groups: two research associates with master’s level social science degrees and the principle investigator. Each has at least 10 years of experience in interviewing and qualitative data collection at this site. The researchers had no relationships with participants prior to their enrollment. Researchers identified themselves as employees of Kaiser Center for Health Research and emphasized what participants shared would be kept confidential. Participants were told that researchers were interested in learning how and why people choose different pain treatment strategies.

We also conducted one time interviews with 25 PCPs in the privacy of their offices. PCPs were distributed, four to five per cell, across three categories of referral frequency to A/C services. Level of referral was determined by comparing an individual’s referral frequencies to those of other health plan providers from January 2008 to June 2010. Low referrers were those at the 0 to 20th percentile, with two to three patients referred to A/C. Moderate referrers were at the 40th to 60th percentile of referrals, with five to 10 patients referred to A/C. High referrers were defined as those at the 80th to 100th percentile of referrals, with at least 15 patients referred to A/C.

We similarly recruited acupuncturists and chiropractors from community settings in Oregon and Southwest Washington who see a high volume of CMP patients from the health plan. One time interviews were completed with eight acupuncturists and six chiropractors in their offices.

Interviews were conducted by one interviewer, who took notes to monitor the interview progress and supplement the transcripts. Focus groups were conducted by pairs of interviewers, with one taking the lead in facilitation, and the other taking notes to supplement the transcripts, tracking the coverage of the focus group guide, the involvement of all participants, and other aspects of group dynamics to insure the greatest involvement across the group. These same staff members using the same guides to conduct patient and practitioner interviews. The guides focused on eliciting information about patients’ and providers’ experiences with and decision-making about using A/C for CMP management. The focus groups and individual interviews with patients explored factors that prompt individuals to seek and continue or discontinue A/C care as well as reasons for seeking care. Open-ended questions were used in interviews with PCPs to elicit referral/treatment practices as well as solicit providers’ knowledge, beliefs, and attitudes about adjunctive care for CMP. Acupuncturists were interviewed and asked to discuss their knowledge, beliefs, and attitudes about adjunctive acupuncture care for CMP conditions and to describe their experiences with the managed care system’s community A/C referral process. Chiropractors were similarly interviewed.

During regular project management meetings, data gatherers provided feedback to the research team. Ensuing discussions provided opportunities for the identification and resolution of any biases or assumptions. Some interview and focus group questions were refined during the first few interviews and focus groups to reduce ambiguities and more reliably elicit the information of interest. Data gathering practices were not designed to reach saturation on any particular topic. Instead, our goal was to capture a range of experiences and perspectives across the participant groups. All interviews and focus groups were audio-recorded and transcribed in preparation for analysis, including standard quality control procedures for transcription accuracy. Participants were not returned copies of their interview transcripts.

### Analysis

Two researchers trained in anthropology oversaw the coding process, which was conducted by two trained coders. The initial codebook was deductively constructed using interview and focus group protocols. As the coding proceeded, additional themes were inductively identified based on repetitions observed in the data [45]. Investigators and coders regularly met to review problematic codes and emerging themes, and refine the codes as necessary. The final codebook contained five broad thematic areas and associated subcodes. Qualitative coding was conducted using Atlas.ti software. Interrater reliability was established through duplicate coding of one out of every six interviews and focus groups, discussion of discrepancies between the coders, clarification of codes where required, and, when necessary, recoding of transcripts. As the coding proceeded, investigators (anthropologist in the lead) and coders regularly met to review problematic codes and emerging themes, and refine the codes as necessary. Participants were not engaged to provide feedback on findings.

The subcode “Opioids and High Dose NSAIDs” was used to capture discussions of prescribing practices, therapeutic uses, and experiences with opioid medications. The analysis described in this paper draws on material from this subcode, as well as material coded under “(A/C) Treatment Benefits,” “(A/C) Treatment Comparisons,” and “Medication Use.”

Where appropriate, we have distinguished between acupuncture and chiropractic care. Discussions of these modalities are largely reported together where issues raised were similar for both (e.g., general processes for referral) but contrasts are pointed out when there were consistent distinctions (e.g., concerns about a therapy).

### Ethics

The Institutional Review Board of Kaiser Permanente Northwest (Federal Wide Assurance # 00002344, IRB #00000405) approved all procedures. Consent forms were reviewed and signed by all participants at the beginning of focus groups or interviews.

## Results

**Fig. 1:**
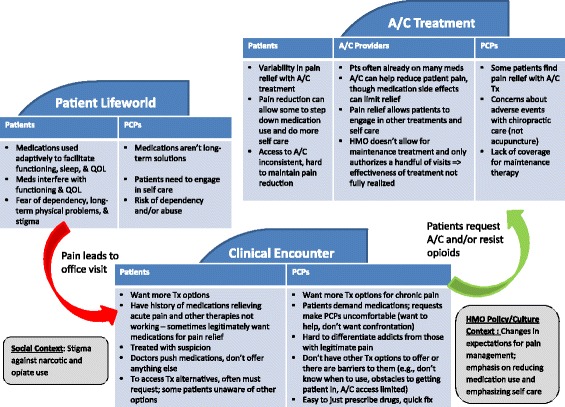
Participants’ views of the complex problem of pain management (abbreviations include: A/C – acupuncture and chiropractic care, PCPs primary care provider, Pts – patients, QOL – quality of life, Tx – treatment, HMO – health maintenance organization

While opioid use was not a focus of the study, it emerged as a dominant theme in the data analysis of participants’ use of A/C for CMP management. For example, PCPs frequently talked about opioids when asked, “What are the biggest barriers and challenges in working with your pain patients?” Discussions about opioids were sorted into four main themes, presented below. First, we provide our participants’ demographic characteristics. Next, we show providers’ attitudes toward opioid use and how all participant groups talked about how opioids were used to manage pain. We highlight the perception that patients may be unwilling to consider options other than opioids. Then we discuss the limited alternative options within the health plan for chronic pain management. We next display how the three groups perceived the potential role of A/C care in pain management. We emphasize how generally they agreed A/C treatments provide a relatively benign therapy that can help patients experience short-term pain relief. However, we note how those perceptions differed based on whether they were talking about acupuncture or chiropractic care. Finally, we explore the complex management of chronic pain and participants’ dissatisfaction with the frequent default to treating pain with opioid medications (Fig. 1—Participants’ views of the complex problem of pain management).

### Participant characteristics

Patient participants for the qualitative study had a mean age of 67.7 years (SD = 12.33) and 70% were female. The racial/ethnic breakdown was 76% white, 8% African American, 2% Native American, 6% other, and 8% unknown/refused to state. Ethnic minorities were specifically oversampled to increase their representation in the study.

Participating PCPs included fairly equal numbers of men (49%) and women (51%) and most identified as white (77%) with 17% Asian respondents and 6% other. Our A/C provider sample included more women (60%) than men (40%) and most identified as white (80%) with 12% Asian respondents and 8% other.

### Options for chronic pain treatment are limited

Participants’ experiences with chronic pain management occurred as clinical guidance and health plan resources were evolving. As one PCP described:

We were all told treat pain, a patient’s pain. You don’t understand why, it’s okay. You have to just acknowledge that they have pain and treat it, treat it, treat it, so they don’t have pain anymore. […] And then the pendulum swung the other way, noting that things like OxyContin were very, very addictive. And there were a lot of patients abusing the medicine, or just having real problems with it. And now, the trend is to really reduce people off the narcotics. [PCP 1]

In response, PCPs said that the health plan introduced several initiatives to reduce and better manage the use of opioid medications. Those included clinician education, periodic urine drug screening of patients, and implementation of written opioid therapy plans to be signed by the patient upon initiation of long-term opioid therapy. These changes positioned PCPs as the gatekeepers and monitors those therapy plans.

Across participant groups, however, respondents reported that despite efforts to discourage and more closely manage opioid use, medications (particularly opioids) were frequently the only option available and respondents from all groups were not satisfied with how they were managed.

The only options I’m given are, you know, like I’m going to be on pain medicines for the next fifty years. [PT 5–4]

I got a ton of drugs thrown at me that I didn’t want. [PT 8–1]

One of the challenges PCPs reported was that while they can refer to pain management, physiatry, and other specialists to help manage complex cases, many CMP patients may already be taking substantial doses of opioids by the time such referrals are placed. PCPs also noted that barriers to other treatment options (including wait times to get into specialty clinics, and patients’ financial constraints), coupled with patients’ desire for quick pain relief and PCPs’ discomfort with potential confrontations with patients should opioid prescriptions be refused, sometimes led PCPs to prescribe opioid medications or prescribe in larger quantities than they might have deemed optimal.

We don’t have very much to offer. […] We don’t have medicines that are really satisfying to give [patients] to help. And, you know, there’s limits on the type of therapy we can put them through. […] Really, all we end up having to offer them is chronic narcotic therapy, which is really dissatisfying to us. [PCP 14]

We keep telling patients, you know, pain medication is only one part. There’s other modalities available. But we’re not reinforcing that. Majority of the time, patients still just get a pain medication. [PCP 2]

A/C providers echoed the perspectives of these two groups:

Mostly when you work with a conventional medical doctor, it is about the prescriptions that the patient is on, because that’s kind of what they do. They know to put them on some kind of medication [CH 48]

You have chronic pain! Oh, yeah? Well, who told you that? Oh, my doctor. Oh, okay. What did they do for you? […] They gave me Vicodin, you know, and they gave me some Flexeril. And they told me just to kind of rest, and it should get better. [CH 45]

### Patients may be unwilling to manage self-care or reduce pain medication

Most PCPs expressed negative attitudes towards opioids for CMP treatment. In describing patients’ trajectories of medication use to control their pain, many articulated a step-wise acceleration that included initial use of over-the-counter medications followed by graduation to opioids and stronger opioids as tolerance increased. While possibly the easiest course of action in a clinic visit, introducing opioids made the situation worse:

Really with chronic pain, the easiest answer is to increase somebody’s narcotic. That’s fast and easy. They think they’re going to get better. They’re out of your office in two minutes. You’re done. But you’ve done a disservice for them long-term. [PCP 22]

PCPs said they would prescribe medications to help patients manage pain, but they stressed the need for patients to participate, engage, and assume responsibility during treatment. PCPs felt that opioid medication was an ineffective, long-term solution that fostered patients’ passivity and reluctance to do anything but take medications for pain management. Patient-driven action was described as the most important aspect of successful pain relief.

PCPs continually pointed to patients’ beliefs in the effectiveness of opioids to reduce pain and the unwillingness of patients to actively manage their condition through lifestyle change as impediments to reducing opioid medications use.

Very often it’s very difficult to find something that works for them. And they end up, I’m hurting, I’m hurting, I’m hurting so much. And they end up wanting to slide into opioids. [PCP 16]

PCPs described unrealistic patient expectations for zero pain, which if used to guide prescribing only led to increasingly powerful medications and doses being prescribed. Some providers referenced a subset of patients who were particularly manipulative and worked very hard to obtain opioid prescriptions. These individuals were characterized as having psychological and dependency issues and using medications inappropriately.

One of our most challenging patients […] we call them doctor shoppers. You know, their last doctor doesn’t want to keep prescribing their opiates, and so they try a new one. [PCP 7]

Such patients might deceptively present themselves to the PCP to get prescriptions to feed their addictions or have mental health problems, such as anxiety, that escalated their sense of pain. These ‘problem’ patients, however, were difficult for clinicians to differentiate from other patients who used the medications appropriately. PCPs expressed discomfort with having to ‘play policeman’ and make these distinctions.

While PCPs generalized about patients being drug seeking, patients’ attitudes toward opioids were much more complex, conflicted, and conditional.

I just need like Vicodin, or a pain med, on occasion. And I don’t like taking it. And I told my doctor that, that I wanted so I could sleep through the night. And now he, well, I’ll give you 10, but it’s got to last. Like he treats me like a drug addict. [PT 1-X]

If you’re going to be able to walk, and you take one pain pill so you can walk and live life, you’re going to do it, even though you may not like it. [PT 2–5]

The immediacy of pain and the functional constraints that pain imposed on patients’ lives could lead them to use medications for a quick remedy, particularly if opioid medications had provided temporary relief in the past. At the same time, side effects of opioid medication (e.g., drowsiness, general unease) sometimes interfered with, rather than supported, functioning and quality of life.

A lot of that medication makes me very drowsy too, so I have a hard time existing healthy-wise, with being like a zombie all the time, because I’m on all this medication. [PT 4–2]

For other patients, opioids did not work for them. The variability of expectations of patients for pain relief and descriptions of compatibility between a person’s body and treatment type were reflected in the difficulty PCPs and A/C practitioners had identifying effective treatment for individual patients.

Patients also discussed the stigma attached to using opioid pain medication. They expressed frustration and discomfort with being treated suspiciously by their PCPs, such as when being restricted to a certain number of pills per month. Patients also illustrated the difficulties of stigma when they tried to legitimize opioid use during focus groups and interviews. For example, many who disclosed that they used opioids were quick to add qualifying statements regarding how they self-limited their use, used only as a response to genuine need (e.g. sleep problems), and never were “buzzed” by their usage. In doing so, these patients differentiated themselves from the stigmatized addicts that PCPs described and explained their usage in terms of how it supported their quality of life and ability to function. While many patients defended their opioid usage, they also expressed concerns about dependence.

A lot of the pain medications are addictive. And that’s the fear. […] I don’t want to be addicted to medication, narcotics. [PT 3–4]

Indeed, across participant groups, long-term opioid use was construed as highly problematic. Providers and patients often used the metaphor of a road to describe how the medication journey, once set upon, was hard to exit. This made medication use of any kind a somewhat daunting prospect, over which patients in particular expressed conflicted feelings. Notably, PCPs almost exclusively framed opioids as problematic because of the risk of dependence.

If [patients] come to me on oxycodone, then I’m not going to be able to get them off of that. [PCP 1]

Usually, after about six months, we progress to providing them with some narcotic pain relief […] even though you tell [patients] it’s a one-way street, even though you tell them it’s kind of the road to perdition, sort of, they feel stuck and they feel like they have to do something to feel better. [PCP 14]

By contrast, patients’ descriptions of fears of dependency were more nuanced and included discussion of the difficult costs and benefits they had to weigh when considering whether or not to take prescription opioids.

Patients, like PCPs, expressed negative attitudes toward medications and some explicitly stated they wished to no longer have to take them. Despite this desire, tapering patients off medications (whether or not they had been used appropriately) was described as very difficult. PCPs cited patients’ resistance to anything other than opioids and their own concerns about harming rapport with their patients by changing prescriptions. While patients described sequencing therapies for pain treatment (e.g., first trying physical therapy, then pain classes, then A/C care), PCPs often expressed frustrations with lack of *timely* patient access to the necessary system resources and with ultimate patient outcomes. Patients also cited the lack of access to other perceived effective therapies or to a structured process for tapering their dosages.

Barriers to more comprehensive and effective treatment, coupled with the immediacy and suffering associated with acute pain exacerbations, sometimes left medications as the only apparent option. One PCP articulated the conundrum this way:

[Chronic narcotic therapy] creates most of our problems in life, in terms of satisfaction with how we work, stuff like that. But you really don’t have many other options, really. […] [Patients are] not really satisfied. Because narcotics just really don’t work. I mean, they work for a while and then they don’t. And then you spend all your time arguing about whether they can have more or not […] Of my top 10 most difficult patients, probably seven of them are people that I just kind of spend my time sort of wrangling about their medications, because I’m trying to keep them safe. And it’s not really their fault. They’re just trying to feel better. And there’s, you know…we can’t get to that place where they feel better and I’m satisfied. [PCP 14]

While PCPs often described patients’ resistance to moving off opioids, this resistance might have less to do with problematic attachment to medications or passive orientations to their role in treatment and more with resistance to experiencing debilitating pain. As one patient explained: “We don’t want to take a pill. We want relief.” [PT 8–4]

### A/C is a relatively benign therapy that can help patients experience short-term pain relief

At several points in patients’ pain and treatment courses, A/C care options are available. In the health plan, medical necessity criteria allow for acupuncture referral for chronic pain conditions, while chiropractic care can be approved for acute, non-radicular back and neck pain. An acute flare of a chronic back or neck condition can be considered an appropriate indication for chiropractic referral by this guideline. PCPs, patients, and chiropractors and acupuncturists all described the potential for A/C care to help reduce and/or replace opioid use. Patients reported:

[Acupuncture] helps me to be me. Doing drugs, I’m not the same person. […] Since seeing an acupuncturist [for last 5 months] I’m completely off every medication I’ve ever been on. [PT 2–9]

I actually had an appointment [with the pain clinic] and was waiting for months. But in the meantime, I had started acupuncture. And so by the time I was seen, I actually didn’t need any additional pain increase meds or anything. [PT 48–6]

While PCPs said:

I’ve seen so many of my patients who really get relief from nothing else, get decent relief from [acupuncture]. […] You see a patient [after they’ve had acupuncture] and they look better. They look relaxed. You don’t see the little furrow between the brows […] they can say to you, oh well, yeah, I have better range of motion. But it’s more than just that. It’s just a sense of, ‘I feel better.’ [PCP 22]

I would be more likely to support and refer [to acupuncture], than to use even some of our most long-standing meds that we’ve used every day in a patient for years. […] it’s that weighing of, what are my options that are relatively safe for this patient? [PCP 20]

However, acupuncturists and chiropractors perceived somewhat different potential impacts of opioid use, patients’ experience, and complicating factors.

When it works with people, it's so nice because they’re not consumed by thinking about their pain. […] it’s great when you get a patient and it lasts a week. You know that they’re getting better at that point. […] It kind of gives them a break to think about something besides the pain. […] When [patients] can stop taking so much medication and have an experience of success with the acupuncture, a number of them are quite elated. Like less Vicodin, you know. [AC 43]

Across groups, while participants described a range of positive experiences with acupuncture, it took time for patients to experience relief, if there was any relief at all. From all groups we heard acupuncture appraised in terms of time:

It took [the acupuncturist] about seven times and it took the pain away. [PT 9–1]

I’m not having that stabbing pain, which I get in my back and in my left hip. And so, you know, then I can go for periods of time where there’s almost no pain. And as long as I go [to the acupuncturist] twice a week that seems to be enough to keep the pain at bay. [PT 30]

I think they fall into kind of two camps. There’s the ones that I went once or twice. And maybe it helped, Doc. I don’t know. But I still hurt. And so there’s kind of a…And then, you know, there’s some that were like, wow, it really helps me. [PCP 7]

I’ll have somebody with chronic pain on a ton of narcotics, VERY excited about their first couple of sessions [of acupuncture] and then they just sort of stop going back. And I’m like, well, you know, why did you stop going back? It seemed like it was…Well, it just stopped working. So the bloom is off the rose, kind of thing. [PCP 10]

I’ve had this thought, especially with tendonitis and plantar fasciitis. I thought to myself, if the doctor had referred them more quickly to acupuncture, it wouldn’t have gone on for so long and they wouldn’t be on the bunch of pain medication that they were on. […] Sometimes those patients that come in are on so many narcotics and have had a number of steroid shots […] So if feels sort of like a drop in the bucket, you know. Like if you could really have them and work with them, over time, you probably could help them to feel better. [AC 43]

The temporariness of the effects and the possible need for follow-up acupuncture sessions often resulted in mixed or uncertain reports of the modality’s effectiveness. All groups believed that acupuncture was a relatively benign therapy that often helped patients experience short-term (at least) pain relief. No one within our sample mentioned adverse effects. The descriptions of their experiences provided information on how acupuncture was used in conjunction with or in place of opioids. However, patients, PCPs, and acupuncturists alike agreed that the result was less satisfactory when patients undergoing acupuncture are already far down the treatment path (i.e., already using opioids and other long-term medications for ongoing pain relief). All groups commented about situations when acupuncture was not a complete solution to CMP.

The only thing that ever really helped me was the morphine that I’m on now. I mean, enough, you know, to function. […] The acupuncture, if I’d have done it longer maybe, but we couldn’t afford to keep doing it. [PT 1–1]

[acupuncture] alleviated the pain. […] I’ve had no negative experience with acupuncture. But I haven’t done it long term for a chronic pain. […] I take pain pills everyday. So, if I have to… Or, if I have the opportunity to go to acupuncture, say a few times a week in hopes that that might help, I would do it [PT 11–8]

I have not ever had acupuncture replace morphine […] people want to do other things, and so they’ll use [acupuncture] with the hope of having to use fewer pain medications. But it never completely replaces that. [PCP 10]

There is an occasional dramatic benefit, but more often transient benefit, and more often no benefit. Patients say, ‘Yeah, I feel better for a day doc, but there was no overall improvement from week to week.’ [PCP 4]

So the elders will be on a ton of meds. And, you know, there’s only so much you can do. It’s side effects on top of side effects on top of side effects. [AC 46]

There’s some cases I couldn’t help, you know, maybe five percent. No more then 10. […] those cases, usually, they are very challenging regardless of who’s treating them, or regardless of what medicine they take. They’ve already gone through a lot in the past. [AC 44]

Comments relating to chiropractic care were also mixed but addressed a slightly different set of issues. Many described the positive ways chiropractic care could impact chronic pain:

When I had the acute pain, when I had that sciatica, [chiropractic] was a lifesaver. It really did make a difference. And I was able to get off of oxycodone and go back to work. [PT 3–1]

Three visits [with the chiropractor] and things were much better. And I could bend and walk, better than taking a pill. [PT 28]

[Patients report outcomes from chiropractic care including] resolution of pain, or significant improvement of pain. Improvement in mobility. So, ability to move around and do the work that they need to do. You know, along with those things comes this feeling of hope. Like, okay, they fixed something. [PCP 1]

[After chiropractic care, patients report] They’re able to go back to work. They’re able to be more mobile. And they don’t need pain medicines for acute pain anymore. [PCP 17]

[The patients] have tried physical therapy. They’ve tried pain management. Nothing has really worked. The docs don’t know what to do. Send them over there. Here, go see a chiropractor. Whether they’re allowed to give our name or what, or it just happens, I’m not sure. But we get a lot of those people. And with them, usually six to 12 visits, we have those people not only feeling better, but back to work. [CH 45]

Typical chronic pain patients will feel immediately better. And occasionally they’ll feel worse. But most of the time they feel better. And I think that surprises them, because they’ve been in this state for so long. […] It’s just changing things up so that their system is starting to work for them a little bit differently. And once that starts happening, blood flows better, circulation is better, attitude is better, mechanics are better. It just starts to work. [CH 48]

As with the experiences described for acupuncture, the patients, PCPs, and chiropractors mentioned the difficulties they encountered maintaining effects. Chiropractors emphasized a successive treatment plan that tapered in frequency.

We do what I call a series of successive approximations. […] They can walk out of here after their first twelve visits or so and go, yeah, I feel pretty good. See ya later. And then they’re back again, and we go through the process over and over and over and over again. [CH 52]

I will often give chronic people anywhere from four to eight treatments, over the course of two months. And then, once every month or two, or once every quarter is a really good approach, in my opinion. [CH 50]

Periodic maintenance visits served, in part, to help correct for existing lifestyle or other complicating factors (e.g., non-ergonomic work environment, obesity) that could erode the benefits experienced early in treatment. By contrast, patients and PCPs frequently focused on shorter duration effects.

Within less than three months [of seeing the chiropractor] the pain was gone. […] I maybe go three or four times a year. And initially, when he was taking care this, it took more than that. [PT 4–10]

When [the health plan] said limited amount [of chiropractor visits], I thought, okay. I probably don’t need six weeks, or whatever, twice a week. I just want them to crack it and get me on my way. [Chuckles] Yeah, just I thought it was going to be one-shot deal. And, it wasn’t. [PT 39]

I think that the benefits from chiropractic are extremely transient. Right? And I’m not really sure…I haven’t had any chiropractic training […] But I don’t think I’ve seen any studies that shows lasting benefit from it. I think the manipulations they do feel as good as cracking any joint. You may get some reflex muscle relaxation, and it’s extremely transient. [PCP 12]

While all groups discussed the transience of pain relief for some patients, that evaluation differed based on whether relief was expected to be long lasting. As with the discussions of acupuncture, when expectations and experience were at odds, this could raise concerns about the quality and efficacy of the received treatment. Unlike with acupuncture, patients and PCPs were more concerned about the quality of chiropractic care patients received, the potential risks posed by adjustments, and the importance of finding the right practitioner.

It took me six different chiropractors until I found a chiropractor that I could actually get relief from. [PT 9–4]

He cracked my neck. And, I immediately knew something was wrong. I wanted to throw up. I thought I was going to pass out. And I had a really bad headache… And it lasted all night long. [PT 49-X]

Occasionally, you hear from someone that the pain is worse (after seeing a chiropractor). I won’t go see him again. He was too rough for me. [PCP 15]

I’ve had a couple (patients say) that they said they felt worse the day after, you know. But then they get better. Or they felt worse, just in general. [PCP 4]

As with acupuncture, however, chiropractic alone was not adequate for pain management.

Right now I’m to a point where I’ll take the occasional Tylenol or Advil, you know, to make it through. But if it’s bad enough, I’ll call my chiropractor. […] I’ve also learned to live with pain too. I’ve learned to know what pain is bad enough that you have to do something. [PT 10–1]

I don’t know if I would go back to a chiropractor. […] The exercises [the chiropractor] gave me still help out a lot. And the [chiropractor] I talked to said, my whole hope is that you won’t really need me anymore. You won’t need to necessarily keep coming back. But you will need other treatments, acupuncture, massage. [PT 3–2]

I’ve had a lot of people say it seemed to help, for awhile. But, most often it doesn’t seem to really create any long-lasting benefit for them. I mean, for people that are clearly on a path of going from injured to getting better, they seem to do a little better through time. [PCP 14]

I would say the ones that end up going, if they get a good one, they really like it. It helps them. Because I think the really good chiropractors also do a lot of physical therapy and ultrasound. And they do a lot of manipulations and good myofascial bodywork for the patient as well. So, I mean, just straight up cracking somebody, I think that’s not going to have as good long-term results. [PCP 17]

I think [chiropractic is] a very appropriate approach to chronic care […] especially if chiropractor emphasizes, you know, exercise, things that the patient can do themselves and not such a passive approach, so the patient doesn’t depend on me to feel better. So you can kind of train somebody to move and keep the restoration of motion going, based on what they do on a daily basis. [CH 50]

I tend to be not at all aggressive with my treatment plans. That goes back to giving people home exercises and stretching, and things like that we talked about prior. Oftentimes, if you have willing people who are willing and capable to do those things, they can get more benefit out of that. [CH 45]

### Managing chronic pain involves multiple contexts, resources, concerns, expectations, and costs and benefits, across time

Many patients expressed dissatisfaction with the lack of alternatives offered for pain management and frequently discussed how they used over-the-counter and prescription medications to cope with pain and to function in everyday life (even if in a limited fashion). The patients also noted fears of becoming addicted or being perceived as addicted to opioid medications. For some, A/C treatment offered a possible solution to this concern. One patient said: “If I was going to get addicted to anything I’d want it to be going to the chiropractor. It seems a lot healthier to me.” [PT 3–4]

Both patients and PCPs reported that a patient’s desire for non-pharmacological options could help encourage referral to A/C practitioners. As one patient described:

I said [to my doctor], I don’t want pain pills. I want to find out why I’m having trouble. I want to get to the root of the problem, and I want to get rid of this pain. And that was the first time that I asked him to see an acupuncturist. [PT 2–1]

This description aligns with what many PCPs told us. For example, a PCP said:

There are patients who really hate drugs. They hate the concept of taking any kind of medication. And so they’re more likely to be open to [acupuncture], and to value it more just for the sole reason that it doesn’t involve taking a medication. [PCP 1]

Mirroring the challenges described by patients, many PCPs expressed frustration with the complexity of treating patients on opioids. As one PCP said:

[Patients using a narcotic drug are] challenging because of the difficulties in separating out issues of dependence versus addiction versus legitimate management of pain. And plus the economic pressures and stuff. And then the issues of abuse and diversion, it’s just like, my God, this is sixteen layers and all I really want to do is treat their pain. [PCP 9]

PCPs continually emphasized problems at the patient level (e.g., addiction, unwillingness to do active self-care), but also frequently mentioned (but ultimately downplayed) other historical and structural barriers to better pain management (e.g., prescribing practices that until quite recently encouraged use of opioids, lack of tools to assist PCPs in managing pain and appropriate timing for different interventions, access issues to alternative treatments, rushed office visits). Despite this, many patients indicated that all they were offered in clinic visits was more medications to manage their pain. Short office visit times likely limit PCP-patient discussions about self-care and facilitate the maintenance of narcotic agents as the ultimate default treatment. As one PCP noted:

We get our little ten-minute per patient, which is so grossly, woefully inadequate amount of time to see a patient. Ten minutes, right, for all your problems. And so nobody wants to take the time to explore things other than drugs for people with chronic pain. [PCP 17]

Almost all PCPs stated that A/C care was not a first-line treatment, so they often would not make referrals to A/C care until the patient had already tried (and failed) several other lines of treatment. Several acupuncturists and chiropractors noted that by the time they saw patients, however, they were already medicated and in a more complicated state than they might otherwise have been if they had been referred earlier in their pain trajectory. As one chiropractor said:

I have to know what the side effects are. And are the side effects why the patient is in here? And therefore, I really can’t do a whole lot about that, other than get them out of some pain. But as long as they’re taking the medication, the joint pain is always going to be there. [CH 48]

The complicated nature of the patients they were seeing, limitations on the number of A/C visits allowed under the referral, and lack of coverage for maintenance therapy created challenges to the outcomes that might be realized.

The incongruence in timing of, expectations for, and plan coverage of A/C treatments were explicit and implicit themes throughout participants’ descriptions of their A/C experiences. Appraisals of and concerns about the modalities sometimes differed, but they contributed to at least some of the frustrations about pain management and the use of opioid medications.

## Discussion

We used qualitative data to explore patient and provider experiences with alternative approaches to opioid pain management. Based on the results presented above, we suggest a new model for thinking about chronic pain. The CMP patient, PCP, and A/C provider narratives illuminate the multifaceted problems and frustrations all three groups face in managing chronic pain. Figure 1 shows how the pieces fit together across contexts. Patients weigh the benefits and harms of treatment routes in light of their daily lives. During a clinical encounter, the perspectives and goals of both patients and their PCPs are again considered and weighed by circumstances and resources. Finally, the experiences and perspectives of patients and their providers are blended with those of A/C carers providing non-pharmacological options for pain management.

Our analyses revealed an array of participant views, impacted by not only PCPs and A/C providers, but also by social context and health policy considerations. While this context is often taken for granted in the research literature, our participant groups described how this occurred locally and provided a specific context for their experiences managing chronic pain. Interview and focus group data uncovered widespread dissatisfaction with opioids among PCPs, patients, and A/C providers. Consistent with this finding, systemic interventions have been implemented at the health plan over the past five to seven years in an effort to stem the tide of opioid misuse. Patients using chronic opioids are now required to sign opioid therapy plans and to submit to periodic urine drug screening. Brief pain assessments are periodically administered to all these patients and the results stored in the electronic medical record. Patients whose daily morphine equivalent intake exceeds set safety limits are being targeted for aggressive tapering, and a dedicated team of pain management pharmacists has been deployed in the primary care clinics to support primary care teams in these efforts. Despite these efforts, however, PCPs expressed dissatisfaction with medication for pain management (see also [46]) and raised questions about the adequacy of resources for primary care to monitor opioid medication use., and many patients still reported that pain medications were the only options provided by their PCPs. Patients pointed to the lack of access to other perceived effective therapies or lack of a structured process for tapering their dosages. While prescribing opioids is increasingly viewed as at odds with best practices for chronic pain management, there remains a lack of feasible alternatives. This discrepancy contributes to discontent across parties, over the course of multiple clinical encounters, and throughout the long course of individuals’ experiences with chronic pain.

There were a range of views as to the appropriate role of A/C care in this context. Those patients who experienced pain relief after A/C treatment indicated that they were no longer debilitated by pain and could engage more fully in recommended lifestyle changes, without the side effects of and long-term dependence on opioids. The lack of coverage for non-acute, maintenance A/C care meant that the ability of A/C to successfully assist patients in managing pain and medication step down may not have been fully realized, though we currently lack the evidence base to guide A/C “maintenance” therapy. Interestingly, Eaves and colleagues [38] found that patients with chronic lower back pain using CAM therapies shifted their expectations for pain relief over the course of CAM treatment and became more accepting of the chronic nature of their pain and their responsibility to engage in self care. That, taken along with patient and A/C provider reports in our own data that self care was a prescribed component of A/C clinical encounters, suggests that there is possibly a role for A/C treatment to reinforce patient acceptance and adoption of self-care measures. This is in contrast to the impression of some PCPs that A/C represents passive modalities that may discourage active, patient self-management practices. Additional studies could examine what within A/C treatment facilitates the adoption of these shifted expectations for pain and self- care, and how PCPs could support that.

Our findings also showed that patients and providers were not always satisfied with the overall effectiveness of A/C treatment. Some of these appraisals were associated with expectations about the timing or duration of relief (sometimes reported as immediate but not long lasting). The number of authorized visits for A/C care was perceived as too few and they occurred over too brief a time span. Suspicion of unfamiliar medical systems and unknown providers was an additional obstacle. There were suggestions in these data that clinical improvements with A/C were less impressive for patients with longer duration of pain and/or who were heavily medicated. These issues require further study.

These findings may also point to a need to better educate both patients and providers about results they can expect in terms of sequencing, time frames, and impacts from A/C treatment, and to a need to tailor treatment benefit structures to these realities. In some cases, patient expectations may be unrealistic. For many patients with long-standing pain, for example, complete elimination of pain will not be possible. Instead, these patients must focus on improving function and quality of life rather than on eliminating pain. Randomized trials suggest that A/C care provides the greatest short-term benefits while the long-term sustainability without continuing treatment is less clear [47]. Unfortunately, for many patients chronic pain is truly chronic.

At the same time, randomized trials suggest that A/C care may have overall efficacy at least as good as conventional medicine [48, 49] that may work by different mechanisms and for different patients. Further, A/C care appears to have fewer adverse effects than many types of conventional treatment. From a clinical research perspective, the potential for A/C care to serve as a non-invasive early alternative to pharmacologic and procedural interventions or as a tool to facilitate the reduction of chronic pharmacotherapy warrants further investigation [50, 51].

Interestingly, patients and PCPs articulated different perspectives of what happened during the clinical encounter, particularly about which group pushed for medications and which opened up possibilities for non-pharmacological alternatives. Patients indicated that while they were concerned about and resistant to initiating opioids due to possible side effects, addiction, and stigma, many had experienced opioids as the only thing that could reduce their pain. Thus, they wanted opioids when experiencing an acute flare and desiring relief and in the absence of other positive treatment experiences. Other researchers have reported similar findings [52, 53]. While no group was happy about it, opioids appear to have become an easy (if ultimately perilous) fallback option during clinical encounters. Previous analyses have described the work and negotiations chronic pain patients need to undertake during clinical encounters [54, 55]. Additional research incorporating observations of clinical interactions, as well as examination of the reasons for patients’ care choices over time, might help to tease out this issue.

Our analysis has several limitations. First, we did not specifically ask participants about opioid medications. A more targeted question-and-answer session could have helped us distinguish nuanced and differing ways patients think and feel about pain medications as a broad class of over-the-counter and prescription medications, and about opioid medications specifically (e.g., [51, 54]). In addition, while we attempted to create homogeneous focus groups by sampling for patients who *had or had not* used the acupuncture or chiropractic benefit through the health plan, we found that across all groups, patients had experiences, at various points and through various means, with acupuncture and/or chiropractic care to treat their pain and other conditions. This factor sometimes made analyzing their experiences by modality difficult. Third, our A/C provider samples were limited to those who received many referrals from the health plan. Having a wider set of acupuncturists and chiropractors, including those not contracted with the health plan, might have provided different perspectives on the treatment of patients with chronic pain and the use of opioid medications. Finally, our codebook was mostly created through deductive processes, whereas the material presented here was largely captured through identification of themes within code reports. Using a more inductive coding approach, such as Grounded Theory, might have allowed for a closer and more systematic reading of our data.

## Conclusion

Our study identified dissatisfaction with using opioids for long-term chronic pain management and the many practical barriers that challenged access to other options from the perspectives of CMP patients, PCPs, and A/C providers. While there was variation among our samples’ experiences with and perspectives of A/C treatment, most of our participants perceived A/C care as helpful in providing short-term pain relief. However, incongruences in timing of, expectations for, and plan coverage of A/C treatments were issues that seemed to limit that potential relief. Our data pointed to the complexity of chronic pain management, particularly given the typical duration of ongoing pain management needs, and the potential benefit for additional study to identify ways to better support clinical decision-making. Finding the best long-term treatment strategies will likely vary depending on the unique patient’s condition, treatment trajectory, expectations, lifestyle, individual values, benefits coverage, and availability of services. While experiences and perceptions of acupuncture and chiropractic care differed along several dimensions, improving access to them would offer providers and patients more options in addressing this complex problem.

Each patient is unique and the persistent theme of the importance of timing in our data point to the complexity of treating individual patients. Finding the best treatment for an individual may depend on his or her condition, where he or she is in his treatment trajectory, and the expectations, lifestyle, individual values, benefits coverage, and availability of services. Moreover, patients typically come in to see their physicians when they are in pain. As our sample exemplified, patients were trying many different things and used A/C care according to their experiences and perceptions of both their pain and the utility and accessibility of different treatments. There is no one best approach, and deciding on which therapies to use and when will depend on many factors in the context of chronic pain management.

## Additional file

Additional file 1: Interview Guide Short Form. A summarized version of the focus group/interview guides used for the data collection. (DOCX 20 kb)

## Abbreviations

A/C: Acupuncture and chiropractic
AC: Acupuncturist
CAM: Complementary and alternative medicine
CH: Chiropractor
CMP: Chronic musculoskeletal pain
HMO: Health maintenance organization
PCP: Primary care provider
PT: Patient
QOL: Quality of life
TX: Treatment
