# Supplementary material for: Provider and patient perspectives on opioids and alternative treatments for managing chronic pain: a qualitative study
Source: BMC Fam Pract. 2017 Mar 24;17:164. doi: 10.1186/s12875-016-0566-0 (PMC5390355; doi:10.1186/s12875-016-0566-0)
Supplement: Additional file 1: — Interview Guide Short Form. A summarized version of the focus group/interview guides used for the data collection. (DOCX 20 kb) [file 12875_2016_566_MOESM1_ESM.docx]

Appendix 1. Interview Guide Short Form

Patient Focus Group / Individual Interview Guide Questions:

1. If a pain treatment “works” or “makes a difference” what has or would you expect to change for you? That is, what would you consider a success?
2. When you received [acupuncture/chiropractic care], what made you seek out [acupuncture/chiropractic care] for treating your pain condition?
3. Overall, has your approach to conventional medical treatment or your conventional medical provider changed at all since seeking [acupuncture/chiropractic] treatment?
4. How have you coordinated your use of [acupuncture/chiropractic care] with other treatments or self-care practices for your pain condition?
5. What are some of the other treatments or self-care routines you’ve tried or been introduced to through Kaiser or your health care providers at Kaiser, including physical therapy? What has made the biggest difference and why?

Primary Care Physician Interview Guide Questions:

1. What types of chronic pain are most common among your patients? Most challenging to treat? What are the biggest barriers and challenges in working with your pain patients?
2. What treatment approaches have you most often taken to working with your pain patients? What approach do you take to working with your most challenging pain patients?
3. Our review of the patients on your panel with chronic musculoskeletal pain suggest that you refer patients for acupuncture/chiropractic care [fairly regularly/occasionally/rarely] When you have made such referrals, why have you chosen to do so?
4. Overall, are there factors that would lead you to NOT recommend or refer a patient for acupuncture/chiropractic care?

Acupuncture/Chiropractor Provider Interview Guide Questions:

1. Can you walk me through how you would go about assessing and treating someone who has come to you wanting help for chronic musculoskeletal pain?
2. Are there particular kinds of people or particular illness histories that you find are especially difficult to treat?
